# Incidence of anemia and predictors among Human Immunodeficiency Virus-infected children on antiretroviral therapy at public health facilities of Bahir Dar City, Northwest Ethiopia: multicenter retrospective follow up study

**DOI:** 10.1186/s12887-022-03168-7

**Published:** 2022-03-03

**Authors:** Gashaw Kerebeh, Yeneneh Ayalew, Demewoz Kefale, Ermias Sisay Chanie, Natnael Moges Misganaw, Dejen Getaneh Feleke, Amare Kassaw, Agimasie Tigabu, Berihun Bantie, Mahlet Tamirat, Teshale Mengesha, Molla Azmeraw, Aklilu Endalamaw

**Affiliations:** 1 Department of Pediatrics and Child Health Nursing, College of Health Sciences, Debre Tabor University, P.O. Box: 272, Debre Tabor, Ethiopia; 2 Department of Pediatrics and Child Health Nursing, School of Health Sciences, College of Medicine and Health Sciences, Bahir Dar University, Bahir Dar, Ethiopia; 3 Department of Adult Health Nursing, College of Medicine and Health Sciences, Debre Tabor University, Debre Tabor, Ethiopia; 4 Department of Pediatrics and Child Health Nursing, College of Medicine and Health Sciences, Dire Dawa University, Dire Dawa, Ethiopia; 5 Department of Nursing, School of Health Sciences, College of Health Sciences, Woldia University, Woldia, Ethiopia; 6 School of Public Health, the University of Queensland, Brisbane, Australia

**Keywords:** Anemia, Children, HIV, Ethiopia

## Abstract

**Background:**

Anemia is one of the common hematological problems among HIV-infected children. It impairs physical functioning, affects the quality of life, increases HIV progression, and decreases survival of HIV-infected children. In Ethiopia, limited studies were conducted on the incidence and predictors of anemia among HIV-infected children on antiretroviral therapy (ART). Therefore, this study aims to assess the incidence of anemia and predictors among HIV- infected children on ART at public health facilities of Bahir Dar City, Northwest Ethiopia.

**Methods:**

An institution-based retrospective follow-up study was conducted among 403 HIV- infected children who have followed at ART clinics in public health facilities of Bahir Dar City from 2010 to 2020. A simple random sampling technique was employed to select the study units. Data was entered using Epi-data version 4.6 and analyzed using STATA 14.0. Cox proportional hazard model assumption was checked graphically and by scaled Schoenfeld residual test. Bivariable Cox-proportional hazards regression model was employed for each explanatory variable to check the association with the outcome variable. Variables with a *p*-value of < 0.2 in the bivariable analysis were candidates to the multivariable proportional hazard model. Cox proportional hazards model was used at a 5% level of significance to identify predictors of anemia.

**Results:**

The overall follow up time was 1587 person–years. The overall incidence density of anemia was 6.87 with 95% confidence interval (CI) = (5.60, 8.16) per 100 person-years. The independent predictors show an association were child age from 0.25 to 5 years adjusted hazard ratio (AHR) = (1.83; 95% CI = 1.22, 2.77), World health organization clinical stage III and IV (AHR = 1.80; 95% CI = 1.22, 2.67), being underweight (AHR = 1.5; 95% CI = 1.01, 2.26), having fair/poor adherence to anti-retroviral therapy (AHR = 1.75; 95% CI = 1.08, 2.85) and zidovidine based anti -retroviral therapy regimen (AHR = 1.72; 95% CI = 1.12, 2.64).

**Conclusion:**

The overall incidence rate of anemia was high compared to other country reports. Age, clinical, and ART-related variables provoked the incidence of anemia. Therefore, a need to emphasize the younger age group, prevent and manage opportunistic infections of WHO clinical stage III and IV, and select and monitor appropriate ART regimen types.

## Background

Hematological abnormalities are common problems in children diagnosed with Human Immunodeficiency Virus [1–3]. Anemia is the most common hematological manifestation in children who are on antiretroviral therapy (ART) [2, 4], which has a significant impact on the quality of life and clinical outcomes unless treated appropriately [5]. Moreover, anemia has serious effects, varying from a physical functioning impairment, psychological distress, and affects the quality of life to an association with disease progression and decreased survival, leading to death [6]. Besides the direct effect of HIV by itself, highly active antiretroviral therapy (HAART) also becomes a cause for anemia like that of Zidovudine (AZT) is known to cause bone marrow suppression [7, 8]. It is a major public health problem affecting an estimated 2 billion people worldwide, with more than 100 million of these anemic children living in Africa [7]. In Africa, the prevalence of anemia after initiation of ART in the pediatrics age group was 54.2% [5]. In Ethiopia, the overall prevalence of anemia among HIV- infected children ranged from 22.3–57.5% [9, 10]. This report also showed that the trend of anemia prevalence among Ethiopian children decreased from 54 to 44% from 2005 to 2011 but increased to 57% in 2016 [10]. Moreover, the incidence of anemia among HIV- infected children was reported in different countries. As reported in Asia, the incidence density rate of severe anemia among children after antiretroviral therapy (ART) initiation was 5.4 per 100 person-years [11]. Similarly, in West Africa, it was 2.47–4.25 per 100 person-year [8]. And in the previous study of Ethiopia, that reports 10.5 per 100 person-years of observation [12].

To prevent and control the incidence of anemia among HIV- infected children, there are different tried solutions at the national and international level. Notably, measuring of serum hemoglobin level before starting ART [13], starting with non-zidovudine (AZT) based ART regimen [14], early initiation of ART [15]. Additionally, start cotrimoxazole prophylaxis for eligible children [16], maintain appropriate nutritional status [17], early diagnosis and management of opportunistic infections (OI) [18], close monitoring of CD4 count and viral load copies [19] has been used as a multi-dimensional approach.

Despite these approaches, there are identified predictors in the previous studies which increases the incidence of anemia like undernutrition [17], low CD4 count [20], taking AZT based ART drug regimen [21], being rural residency [22], advanced disease stage and OI [23].

Even though post ART anemia increases in children, studies on incidents and predictors of anemia among children initiating ART were limited; most studies were conducted on the prevalence of post ART anemia in different areas of the World. Similarly, in our country Ethiopia, data on incidents of anemia and predictors among children who are on ART is limited. Therefore, this study aims to assess the incidence of anemia and predictors among HIV- infected children at public health facilities of Bahir Dar City, Northwest Ethiopia.

The results found from this study provide valuable information for policymakers, clinicians, and researchers to enhance decision-making and planning of appropriate interventional strategies to reduce the incidence of anemia in HIV-infected children.

## Methods and materials

### Study design, setting, and period

An Institution-based retrospective follow-up study was conducted at public health facilities of Bahir Dar City, Northwest Ethiopia. Bahir Dar City has located 565 km from Addis Ababa, the capital City of Ethiopia. According to the 2020 Bahir Dar City administration Health Department report, the total population is 389,177. Of these, 147,983 were children less than 15 years. The City has three governmental Hospitals (one specialized teaching Hospital, one comprehensive specialized referral Hospital, one primary Hospital) and ten governmental health centers. From the governmental health facilities, two Hospitals and eight Heath centers provide ART services for HIV- infected children. In the above health facilities, there are a total of 1,117 HIV- infected children who started ART since ART service started, and there are a total of 763 live HIV- infected children on ART follow up till now. Data were extracted from those children who started ART follow-ups between September 1, 2010, and December 31, 2020.

### Source population

All HIV infected children < 15 years of age started ART at public health facilities of Bahir Dar City ART clinic.

### Study population

All HIV-infected children < 15 years of age started ART at public health facilities of Bahir Dar City ART clinic from September 1, 2010, to December 30, 2020.

### Eligibility criteria

#### Inclusion criteria

All HIV-infected Children < 15 years of age who took ART at least one month within September 1, 2010, to December 30, 2020.

#### Exclusion criteria

Children who had anemia at baseline and transferred from other health facilities were excluded from the study. Additionally, children with incomplete chart recording at baseline and during the follow-up period, especially important variables like age, sex, serum HGB level, weight, height, ART regimen, date of ART initiation, and date event or censored reported excluded from the study.

### Sample size determination and sampling procedure

The sample size was determined using Log-rank test through open STATA version 14.0 statistical software with the assumptions of 95% CI, exposed to unexposed ratio 1:1, the margin of error 5%, power 80%, and 10% for incomplete chart records, which was calculated by taking significantly associated predictor for the incidence of anemia (being male sex) 4.6% and adjusted hazard ratio (AHR:2.36), from studies conducted on incidence and predictors of severe anemia in Asian HIV-infected children [11]. First, all public health facilities providing ART services in Bahir Dar City are identified. Then, the lists of children who have started ART during the study period were obtained from their registration book and electronic database in each health facility. Next, the sampling frame was constructed by adding from each health facility among medical records of children on ART. Finally, from the constructed sampling frame, HIV-infected children were selected randomly by using a computer-generated simple random sampling technique.

**Fig. 1:**
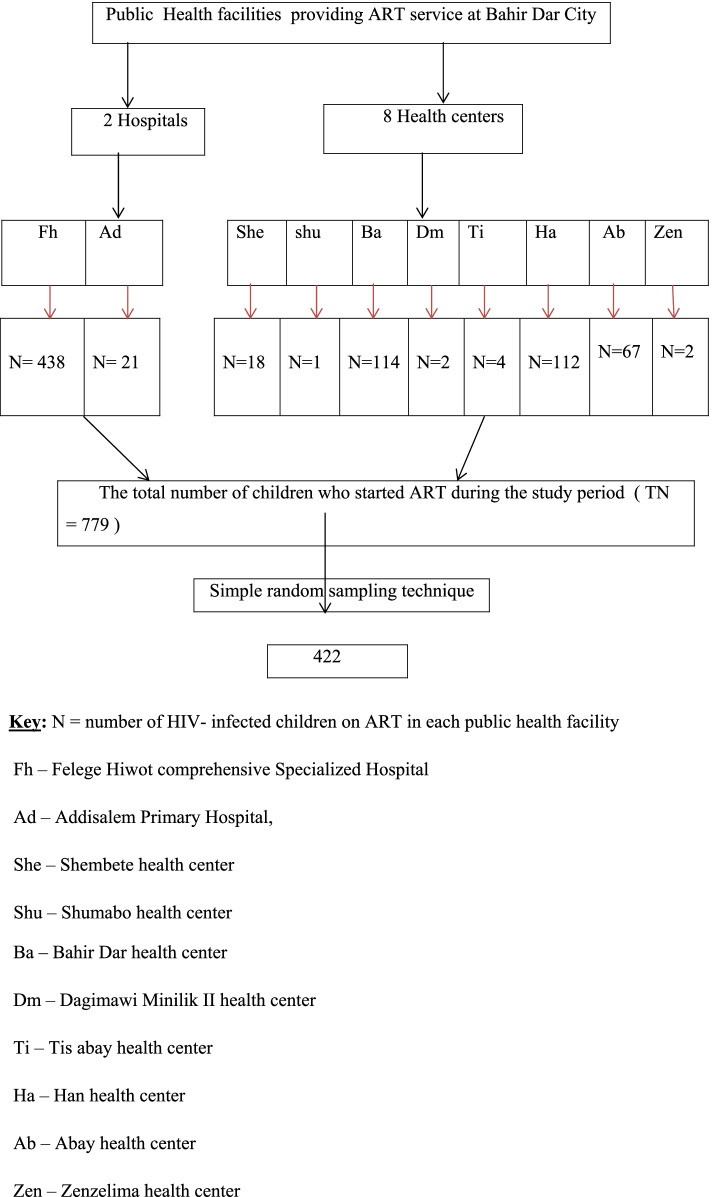
Sampling procedure for incidence and predictors of anemia among HIV- infected children on ART at public health facilities of Bahir Dar City, Northwest Ethiopia, from 2010 to 2020

The total sample size of 422 HIV- infected children were selected from the health facilities (Fig. 1).

### Study Variables

The dependent variable was the incidence of anemia. The independent variables include socio-demographic variables of the child (age, sex, residence, family size, parental live status). The socio-demographic variables of caregiver were age, HIV status, occupation, educational status, relation with the child, marital status. Clinical, laboratory and treatment-related characteristics (WHO clinical stage, CD4 count, Serum Hemoglobin level, Nutritional status, past TB history, initial ART regimen, time of ART initiation, regimen change, treatment duration, level of adherence, OI prophylaxis, current TB treatment, medication side effect, and viral load copies).

### Operational definitions

Event: the occurrence of anemia during the follow-up time.

Anemia: anemia is defined as a hemoglobin level less than 11 g/dl for children < 5 years old, < 11.5 g/dl for children 5–11.9 years old, and < 12 g/dl for children 12–14.9 years [24].

Time-to-event: Defined as the time interval between the date when follow-up started and the occurrence of anemia.

Censored: when HIV-infected children withdraw, lost, transfer out, dead or the study period ends before HIV-infected children develop anemia.

ART initiation: it can be explained as early initiation or late initiation. Early initiation: defined as ART initiation within seven days of HIV diagnosis provided no contraindications. Late initiation: defined as ART initiation after seven days of HIV diagnosis.

Nutritional status: classified as Well-nourished, mild malnourished, moderately malnourished, and severely malnourished [25].

Well nourished: according to WHO growth curve weight/age < 1 & ≥ -1 z score, height/age < 1 & ≥ -1 z score, weight/height < 1 & ≥—1 z score.

Mild under nutrition: according to WHO growth curve weight/age < -1, ≥ -2 z score, height/age < -1, ≥ -2 z score, weight/height < -1, ≥ -2 z score.

Moderate under nutrition: according to WHO growth curve weight/age < -2, ≥ -3 z score, height/age < -2, ≥ -3 z score, weight /height < -2, ≥ -3 z score.

Severe under nutrition: according to WHO growth curve weight/age < -3 z score, height/age < -3 z score, weight/height < -3 z score.

Past TB history: a child who had TB and completed the anti-TB treatment before enrolment in this study.

ART Adherence: is defined as "good" if the child took ≥ 95% ( missing one from 30 doses or three out of 60 doses), "fair" if the child took 85- 94% ( missing 2–4 doses out of 30 doses or 4–9 from 60 doses or "poor "if the child took < 85% ( missing ≥ 5 doses from 30 doses or > 10 from 60 doses during follow up [26].

### Data collection tool and procedure

Data were collected from HIV-infected children charts using a prepared and pre-tested data extraction structured checklist, adapted from WHO ART follow-up forms and intake form [27] included by reviewing related literature. Data were collected regarding HIV-infected children's socio-demographic variables, clinical, laboratory, and treatment-related variables. Three bachelors of science (BSc) nurses who have experience working in ART clinics and took comprehensive ART training were selected for data collection. One BSc nurse who has a comprehensive ART training certificate was selected for supervisor. The charts were retrieved using medical registration numbers from the computer database.

### Data quality assurance

A pre-test was conducted at Bahir Dar Health center among 22 medical records of children on ART using the prepared checklist before 02 weeks of the actual data collection period to check the consistency of the checklist and availability of study variables. Two days of training were provided for data collectors, how to review the documents and extract data from medical records. And for one supervisor, how to supervise the entire data collection process. The filled formats were checked for completeness by the data collectors, supervisor, and principal investigator each day, and data cleaning was done during data collection and analysis time. Once data were extracted from patient charts, it was coded to avoid duplication.

### Data processing and analysis

The collected data were coded and entered into EPI data version 4.6, and it was exported into STATA version 14.0 for cleaning and analysis. WHO anthro and WHO anthroplus software was used to calculate the Z-score of weight for age, body mass index for age, height for age, and height for weight to determine the nutritional status of children. Descriptive and summary statistics were computed to determine frequencies and proportions. Multicollinearity was checked using correlation coefficient (the value was less than 0.6) and variance inflation factor (the average VIF was 1.45).

Cox proportional hazard model assumption was checked graphically with the log–log plot of survival estimate and by scaled Schoenfeld residual test (*p*-value greater than 0.05 was met the assumption). The overall model fitness was checked using the cox–Snell residual test. The incidence rate of anemia was calculated using person-years of observation as a denominator for the entire study period. Kaplan Meier survival curve was used to estimate the anemia-free probability time and survival function estimation among different groups of categorical explanatory variables to compare with the support of log-rank test.

The bivariate Cox-proportional hazards regression model was employed for each explanatory variable to check the association with the outcome variable. Variables with a p-value of < 0.2 in the bivariable analysis were candidates to the multivariable proportional hazard model. A 95% CI of hazard ratio (HR) was computed, and variables with a p-value less than 0.05 in the multivariable model were considered significantly associated with the dependent variable. Missing values were identified in four variables, and the percentage was ranged from 1.2% to 3.5% so, multiple imputations were implemented to fill the missed values.

### Ethical considerations

Ethical clearance was obtained from the Ethical Review Board of Bahir Dar University College of Medicine and Health Sciences with IRB number (CMHS/IRB 01–008). A supporting letter was obtained from each selected health facility general manager, health center head, and coordinator. Information in the data extraction tool was anonymous. Files of entered data in the software and the final result of the study were protected with a password. The confidentiality of information was kept throughout the entire study process, and the information was used only for the study purpose.

## Results

### Baseline Socio-demographic characteristics of children on ART

A total of 422 charts were reviewed. Of which, 403 medical records of HIV- infected children on ART were included in the analysis that provided a completeness rate of 95.5%. The median age of HIV- infected children during ART initiation was eight years with IQR [5, 10]. More than half (53.35%) of the participants were males. The majority (82.13%) of children were from an urban area, and two-thirds (66.50%) of the children had a family size of less than three (Table 1).

**Table 1:** Anemia incidence density rate stratified by baseline Socio-demographic characteristics of children on ART at public health facilities of Bahir Dar City, Northwest Ethiopia from 2010 to 2020

Variable	*N* = 403 (%)	Anemia *N* = 109	Censored *N* = 294	Person-Years	IDR/100 PYO
Sex					
Male	215 (53.35)	60	155	850.56	7.05
Female	188 (46.65)	49	139	737.19	6.64
Age of child					
0.25—≤ 5	120 (29.78)	42	78	428.87	9.79
6–10	186 (46.15)	43	143	788	5.46
11–14	97 (24.07)	24	73	370.88	6.47
Residence					
Urban	331 (82.13)	93	238	1300.48	7.15
Rural	72 (17.87)	16	56	287.26	5.57
Family size					
1–3	268 (66.50)	67	201	1035.52	6.47
4–7	135 (33.50)	42	93	552.22	7.60

### Baseline socio-demographic characteristics of caregiver and parents

More than half (62.03%) of the HIV- infected children's parents, both mother, and father, were alive, And almost more than two-thirds (90.07%) of the HIV- infected children were living with their parents. Nearly greater than half (62.03%) of parents were married. More than one-third (44.67%) of HIV- infected children, parents both father and mother were HIV positive (Table 2).

**Table 2:** Anemia incidence density rate stratified by baseline socio-demographic characteristics of caregiver and parents information at public health facilities of Bahir Dar City, Northwest Ethiopia from 2010 to 2020

Variable	*N* = 403 (%)	Anemia *N* = 109	Censored *N* = 294	Person-Years	IDR/100 PYO
Age of caregiver					
15–30	158 (39.21)	47	111	572.44	8.21
31–45	222 (55.09)	55	167	927.90	5.93
46–65	23 (5.7)	7	16	87.41	8.00
Parental live status					
Both a live	250 (62.03)	67	183	1014.50	6.60
Only mother a live	76 (18.86)	20	56	297.47	6.72
Only father a live	46 (11.41)	16	40	157.62	10.15
Both dead	21 (5.21)	4	17	93.37	4.28
Unknown	10 (2.48)	2	8	24.77	8.07
Relation of caregiver					
Parent	363 (90.07)	102	261	1417.13	7.19
Guardian	40 (9.93)	7	33	170.62	4.10
Educational status of the caregiver					
Unable to read and write	92 (22.83)	22	70	345.31	6.37
Primary education	117 (29.03)	34	83	478.56	7.10
Secondary education	78 (19.35)	27	51	300.24	8.99
College/University level	116 (28.78)	26	90	463.64	5.60
HIV status					
Both parents positive	180 (44.67)	46	134	753.82	6.10
Mother positive	128 (31.76)	37	91	519.57	7.12
Father positive	30 (7.44)	13	17	78.84	16.48
Father negative	4 (0.99)	0	4	27.49	0
Caregiver positive	2 (0.50)	0	2	8.38	0
Caregiver negative	15 (3.72)	4	11	53.26	7.51
Unknown	44 (10.92)	9	35	146.37	6.14
Marital status of the caregiver					
Single	31 (7.69)	7	24	110.33	6.34
Married	250 (62.03)	68	182	992.19	6.85
Divorced	24 (5.96)	12	12	98.19	12.22
Widowed	98 (24.32)	22	76	387.03	5.68
Occupational status of the caregiver					
Government employee	111 (27.54)	25	86	445.77	5.60
Farmer	35 (8.68)	9	26	127.98	7.03
Merchant	86 (21.34)	27	59	307.23	8.78
House wife	77 (19.11)	20	57	305.92	6.53
Daily worker	59 (14.64)	20	39	243.10	8.22
Self-employee	35 (8.68)	8	27	157.72	5.07

*IDR* Incidence density rate, *PYO* Person-years of observation

### Baseline Clinical, Laboratory and ART information of children on ART

Regarding opportunistic infections at baseline, (53.85%) of HIV-infected children were experienced opportunistic infections. Of which, (19.8%) had recurrent upper respiratory tract infections followed by herpes zoster (14.7%), tuberculosis (14.7%), and bacterial pneumonia (13.8%). More than two-thirds (84.37%) of HIV-infected children took cotrimoxazole prophylaxis during ART initiation. Related to the initial ART regimen started for the participants, 37.46% of children initiated with a combination of AZT-3TC-NVP. Of the total HIV- infected children, about 45.66% of children were stunted, and 42.18% of participants were underweight (Table 3).

**Table 3:** Anemia incidence density rate stratified by baseline Clinical, Laboratory, and ART information of children at public health facilities of Bahir Dar City, Northwest Ethiopia from 2010 to 2020

Variable		*N* = 403 (%)	Anemia *N* = 109	Censored *N* = 294	Person-Years	IDR/100 PYO
WHO clinical stage						
Stage I / II		279 (69.23)	63	216	1114.03	5.65
Stage III / IV		124 (30.77)	46	78	473.71	9.71
CD4 count						
> 350 cells/µl		242 (60.05)	63	179	916.58	6.87
350 – 200 cells/µl		95 (23.57)	26	69	409.28	6.35
≤ 200 cells/ µl		66 (16.38)	20	46	261.88	7.64
OI at baseline						
Yes		217 (53.85)	66	151	903.76	7.30
No		186 (46.15)	43	143	683.98	6.28
Cotrimoxazole Prophylaxis						
Yes		340 (84.37)	92	248	1405.16	6.54
No		63 (15.63)	17	46	182.59	9.31
History of past TB						
Yes		15 (3.72)	1	14	57.322	1.74
No		388 (96.28)	108	280	1530.42	7.05
Isoniazid prophylaxis						
Yes		97 (24.07)	22	75	312.06	7.04
No		306 (75.93)	87	219	1275.69	6.81
ART regimen started						
AZT-3TC-NVP		151 (37.46)	53	98	603.04	10.62
d4t-3TC-NVP		60 (14.88)	21	39	260.56	8.05
d4t-3TC-EFV		14 (3.47)	1	13	88.65	1.12
AZT-3TC-EFV		95 (23.57)	22	73	393.52	5.59
TDF-3TC-EFV		26 (6.45)	2	24	104.82	1.90
ABC-3TC-LPV/r		20 (4.96)	6	14	35.80	16.75
ABC-3TC-EFV		11 (2.73)	1	10	44.70	2.23
ABC-3TC-NVP		11 (2.73)	3	8	40.08	7.48
TDF-3TC-DTG		1 (0.25)	0	1	0.89	0
ABC-3TC- DTG		8 (1.99)	0	8	8.71	0
ABC- 3TC-EFV		1 (0.25)	0	1	7.66	0
AZT-3TC- LPV/r		1 (0.25)	0	1	0.33	0
ABC- 3TC- NVP		1 (0.25)	0	1	2.74	0
TDF- 3TC- NVP		3 (0.74)	0	3	18.14	0
Time of ART initiation						
Early initiation		72 (17.87)	17	55	188.87	9.00
Late initiation		331 (82.13)	92	239	1398.87	6.57
Height/length for age (< 15 years)	Normal	219 (54.34)	52	167	910.93	5.71
	Stunted	184 (45.66)	57	127	676.81	8.42
Height/length for wieght (≤ 5 years)	Normal	93 (77.50)	30	63	363.19	8.25
	Wasted	27 (22.50)	9	18	97.46	9.23
Weight for age (< 10 years) or BMI for age (≥ 5 years)						
Not under weight		233 (57.82)	52	181	968.98	5.36
Under weight		170 (42.18)	57	113	618.76	9.21

*IDR* Incidence density rate, *PYO* Person years of observation

### Follow up laboratory and ART information

The majority (88.09%) of children had a good ART adherence during their follow-up. Two-thirds (66.75%) of HIV-infected children have changed their original ART regimen. The major (52.79%) reason for changing the regimen was the availability of new drugs (Table 4).

**Table 4:** Anemia incidence stratified by follow up laboratory and ART information of children on ART at public health facilities of Bahir Dar City, Northwest Ethiopia, from 2010 to 2020

Variable	*N* = 403 (%)		Anemia *N* = 109	Censored *N* = 294	Person- Years	IDR/100 PYO
Viral load						
≤ 1000 copies/ml	333 (82.63)		88	245	1331.15	6.61
> 1000 copies/ml	70 (17.37)		21	49	256.59	8.18
Level of ART adherence						
Good	355 (88.09)		88	267	1413.24	6.22
Fair/Poor	48 (11.91)		21	27	174.50	12.03
Side effect						
Yes	51 (12.66)		29	22	151.51	19.14
No	352 (87.34)		80	272	1436.23	5.57
Regimen change						
Yes	269 (66.75)		85	184	1158.64	7.33
No	134 (33.25)		24	110	429.10	5.59
Reason for regimen change (*n* = 269)						
Toxicity/side effect	67 (24.91)		32	55	250.46	12.77
New drug available	142 (52.79)		36	106	611.13	5.89
Dug stock out	30 (11.15)		10	20	149.24	6.70
New tuberculosis	3 (1.12)		0	3	15.09	0
Treatment failure	27 (8.55)		7	20	108.39	6.45
Others	4 (1.49)		0	4	24.3	0
Follow up time in month						
1–6	64 (15.88)	48		16	184.25	26.05
6–12	24 (5.96)	10		14	164.14	6.09
12–24	42 (10.42)	15		27	293.16	5.11
24–36	42 (10.42)	12		30	255.14	4.70
> 36	231(57.32)	24		207	714.15	3.36

Others include patient preference and not recorded the reason for regimen change, *IDR* Incidence density rate, *PYO* Person years of observation

### Incidence of anemia during follow up period

A total of 403 HIV- infected children were followed for the overall follow-up period of 1587 person-years of observation with a minimum of one month and a maximum of 123 months. From 403 total study participants, 226 (56.08%) were on ART at the end of the study period, 14 (3.47%) were lost to follow up, 47 (11.66%) were transferred out to other health facilities, 7 (1.74%) have died, and the rest 109 (27.05%) were developed anemia.

**Fig. 2:**
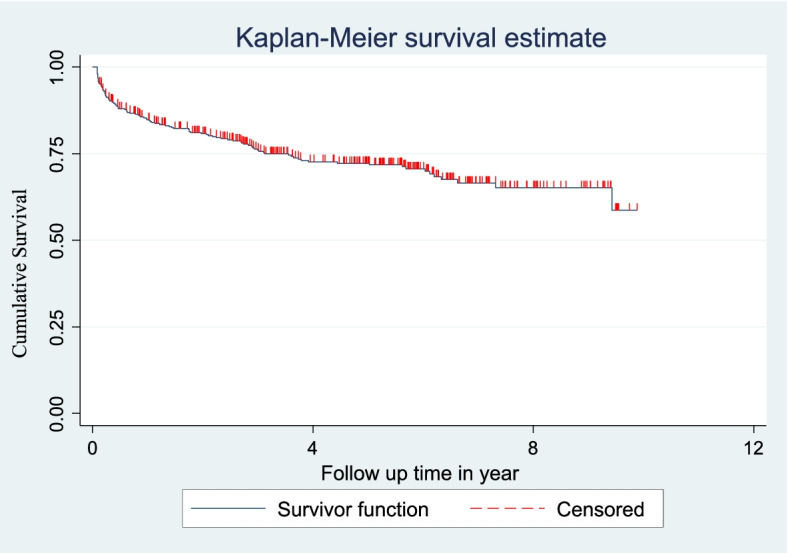
The overall Kaplan Meier survival estimate of HIV- infected children on ART at public health facilities of Bahir Dar City, Northwest Ethiopia, from 2010 to 2020

The overall incidence density rate (IDR) during the cohort was 6.87 (95% CI = 5.60, 8.16) per 100 person-years of observation and the cumulative proportion was 27.05% (95% Cl = 22.91, 31.61). The cumulative probability of anemia free at 6, 12, 24, 36 and 123 months of ART initiation was 88%, 84%, 80%, 76%, and 58%, respectively. The highest anemia incidence density rate among children on ART was 26 (95% CI = 19.63, 34.56) per 100 person-years of observation during the first 6 month of follow up and decreased to 6 (95% CI = 3.27, 11.32), 5 (95% CI = 3.08, 8.48), 4.7 (95% CI = 2.67, 8.28) and 3.3 (95% CI = 2.25, 5.01) per 100 person-years of observation in 12-month, 24-month, 36-month and > 36 month, respectively. Anemia free probability by the end of the follow up was 58% (95% CI = 51.30, 63. 36) (Fig. 2).

### Log-rank test result comparison on different categorical variables

A Log-rank test was performed to test the equality of survival curves of different categorical explanatory variables. The test statistics showed a significant difference in survival function in different categorical explanatory variables.

**Fig. 3:**
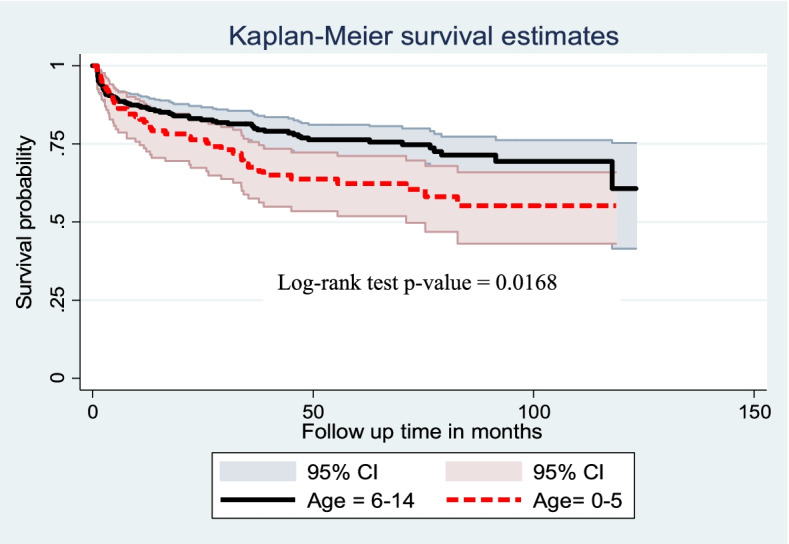
Kaplan Meier survival curve of anemia free survival proportion based on age among children on ART at public health facilities of Bahir Dar City, Northwest Ethiopia, from 2010 to 2020

In this study, a child aged 0.25 to 5 years had lower survival status than those greater than five years of age. The overall survival of those with age 0.25 to ≤ 5 years and > 5 years are found to be 55% and 61%, respectively (Fig. 3).

**Fig. 4:**
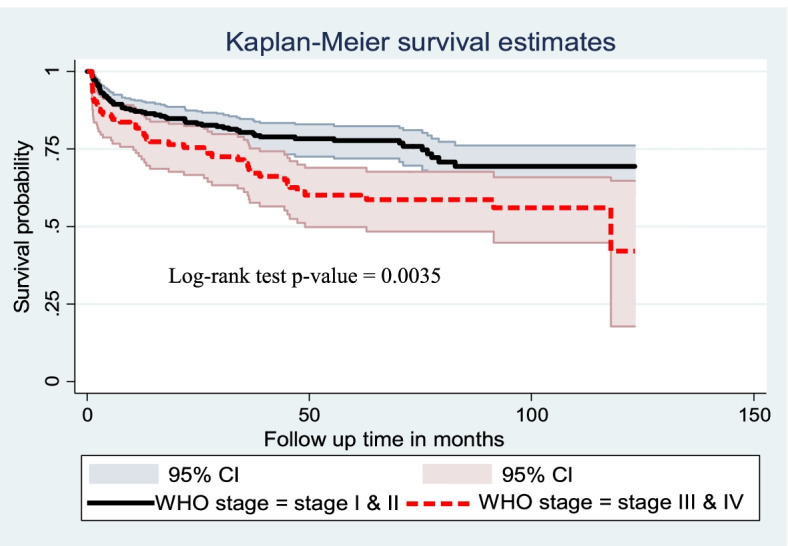
Kaplan Meier survival curve of anemia free survival proportion based on WHO clinical stage among children on ART at public health facilities of Bahir Dar City, Northwest Ethiopia from 2010 to 2020

In addition, children with WHO clinical stages III & IV had lower survival times as compared to children with WHO clinical stages I & II. The median survival time of anemia-free children with stage III & IV was 117 months. The overall survival of children with WHO clinical stage III & IV and WHO clinical stage I & II were 43% and 69%, respectively (Fig. 4).

**Fig. 5:**
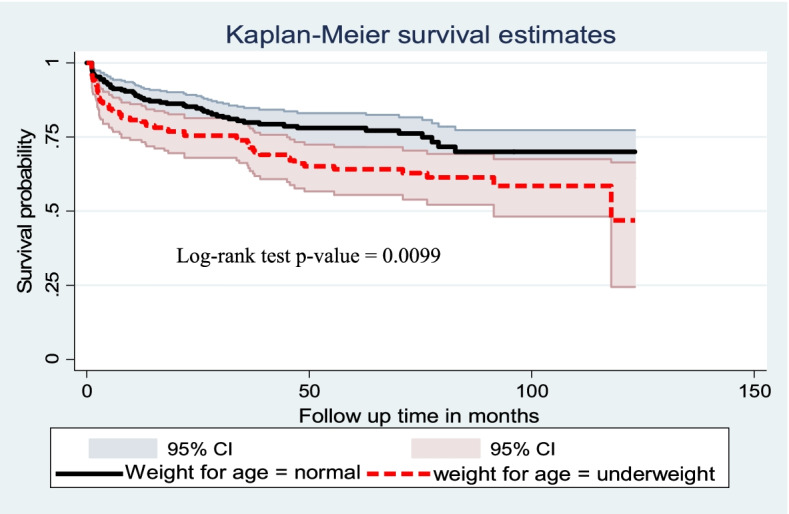
Kaplan Meier survival curve of anemia free survival proportion based on nutritional status among children on ART at public health facilities of Bahir Dar City, Northwest Ethiopia, from 2010 to 2020

Similarly, children with underweight had lower survival status than children with not underweight. The median survival time of anemia-free for underweight children was 117 months, and the overall survival of underweight children and not underweight children was 47% and 70%, respectively (Fig. 5).

### Testing the model goodness of fitness

**Fig. 6:**
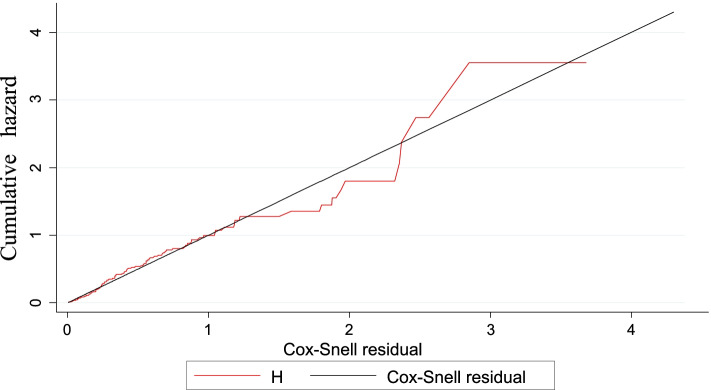
Cox Snell residual test to the goodness of fit of Cox proportional hazard model for children at public health facilities of Bahir Dar City, Northwest Ethiopia from 2010 to 2020

The goodness of fit test for cox- proportional hazard regression model was done by cox -Snell residual test in which the hazard function curve follows the 45-degree line closely, as we can confirm from the graph below (Fig. 6).

### Predictors of Anemia among children on ART

The temporal relationship between the baseline variables and the risk of anemia on HIV- infected children on ART was analyzed using the Cox proportional hazard regression model. In bivariable analysis, the Cox proportional hazard regression model showed the age of the child, the relation of caregiver to a child, marital status of the caregiver, WHO clinical stage, height for age, weight for age / BMI for age, history of past TB, ART adherence and ART regimen were identified as predictors of anemia. In the multivariable analysis, only child's age, WHO clinical stage, weight for age / BMI for age, ART adherence, and ART regimen were predictors of anemia.

This study finding showed that the hazard of anemia in children whose age was between 0.25–5 years was 1.83 (95% CI = 1.22, 2.77) times higher than children whose age was > 5 years. Similarly, a hazard of anemia in children with WHO clinical stage III and IV had increased 1.8 (95% CI = 1.22, 2.67) times compared to children with WHO clinical stage I and II. In addition to this, being underweight increased the hazard of anemia by 1.50 (95% CI = 1.01, 2.26) times compared to children who are not underweight. Children with fair/poor ART adherence have increased the hazard of anemia by 1.75 (95% CI = 1.08, 2.85) times compared with children who had good ART adherence levels. Furthermore, the hazard of anemia in children who started AZT- based ART regimen was 1.72 (95% CI = 1.12, 2.64) times higher than those who started non-AZT-based ART regimen (Table 5).

**Table 5:** Bivariable and multivariable cox regression analysis for predictors of anemia in children on ART at public health facilities of Bahir Dar City, Northwest Ethiopia from 2010 to 2020

Variable	Category	Status		CHR (95% CI)	AHR (95% CI)	P–value
		Censored (*n* = 294)	Anemia (*n* = 109)			
Age of child	6–14 years	216	67	1	1	
	0.25—≤ 5 years	78	42	1.59(1.08, 2.34)	1.83(1.22, 2.77)	0.004
Relation of caregiver	Parent	261	102	1.68(0.78, 3.63)	0.53(0.19, 1.43)	0.212
	Guardian	33	7	1	1	
Marital status	Single	24	7	1	1	
	Married	182	68	1.11(0.51, 2.43)	0.71(0.25, 1.99)	0.520
	Divorced	12	12	2.08(0.81, 5.29)	1.39(0.46, 4.22)	0.551
	Widowed	76	22	0.95(0.40, 2.23)	0.66(0.23, 1.89)	0.446
WHO stage	Stage I & II	216	63	1	1	
	Stage III & IV	78	46	1.75(1.19, 2.56)	1.80(1.22, 2.67)	0.003
Wt. for age / BMI for age	Not underweight	181	52	1	1	
	Under weight	113	57	1.63(1.12, 2.37)	1.50(1.01, 2.26)	0.048
Height for age	Normal	167	52	1	1	
	Stunted	127	57	1.39(0.95, 2.03)	1.26(0.83, 1.90)	0.263
History of past TB	No	280	108	1	1	
	Yes	14	1	4.04(0.56,28.95)	4.05(0.56, 29.2)	0.165
ART adherence	Good	265	88	1	1	
	Fair/ poor	29	21	1.88(1.16, 3.03)	1.75(1.08, 2.85)	0.024
ART regimen Stared	Non -AZT based regimen	265	88	1	1	
	AZT based regimen	172	77	1.52(1.01, 2.29)	1.72(1.12, 2.64)	0.013

Statically significant at *p*- value < 0.05

## Discussion

This study's overall incidence density rate was higher than the studies conducted in Asia (Cambodia, India, Indonesia, Malaysia, Vietnam, and Thailand), reported 5.4 per 100 person-years of observation [11]. And West Africa (Benin, Burkina Faso, Cote d'Ivoire, Gambia, Ghana, Mali, and Senegal) revealed 2.47 per 100 children-years observation in those on Zidovudine containing regimen versus 4.25 in those non-zidovudine based regimens [8]. This difference might be due to differences in ART drug regimen and multi-centeredness. Because both previous studies were done with multi-center at country level, but this study was done the multicentered at the health facility level. Moreover, the previous research reported only severe anemia incidence, but the current study reported the incidence of all types of anemia. In the study conducted in West Africa, the follow-up time included only the duration of first-line antiretroviral therapy, but this study included the follow-up time after first-line ART was changed to the second-line/third-line ART drug. On the other side, the overall incidence density rate of the current study was lower than the study conducted in Gondar, Ethiopia, 10.5 per 100 person-years of observation [12].

The current study showed that the hazard of anemia in children whose age between 0.25—5 years was higher than children whose age was greater than five years. This study is supported by the study conducted in West Africa, India, and Uganda [5, 8, 23]. Due to that, children in this age group had an increased micronutrient requirement for growth and a higher frequency of gastrointestinal infections. Since the disease progression is fast, they are more at risk for malnutrition and opportunistic infections. In addition, the most common cause of anemia in under-five children is low consumption and absorption of iron-rich foods (i.e., meat and meat products). These conditions most often lead to iron deficiency anemia, accounting for approximately half of all anemia cases globally [9, 23, 28].

Similarly, the hazard of anemia in children with an advanced WHO clinical stage of disease during ART had increased 1.8 times as compared to their counterparts.

This study is supported by the study conducted in India and Uganda [5, 23]. It can be explained that being in the advanced stage of the disease causes compromised immunity, which leads to increased viral multiplication and higher loads of opportunistic infections.

This causes anemia through increased cytokine-mediated myelosuppression and a higher burden of comorbidities [5]. This study showed that being underweight at the baseline increased the hazard of anemia by 1.50 times compared to children who are not underweight. It is in line with the study done in Asia, West Africa, and a previous study in Ethiopia [8, 11, 12] because children with underweight will have intestinal mal-absorption and micronutrient deficiency, like iron, folic acid, vitamin A, and vitamin B12 [29, 30] which leads to anemia.

In this study, children having fair/poor ART adherence during follow-up time increased the hazard of anemia by 1.75 times compared to children who had good ART adherence levels. This statically associated result is supported by the study conducted in Ethiopia [31]. The possible explanation for this might be those children who had fair/poor ART adherence are at a greater risk of high viral load duplication, suppression of immunity, fast progression of the disease, development of opportunistic infections, drug resistance. And further clinical deterioration may occur, resulting in anemia via cytokine-mediated myelosuppression [32, 33].

Furthermore, the current study showed that the hazard of anemia in children who started AZT- based ART regimen during initiation was 1.72 times higher than those who started non-AZT-based ART regimen. It is supported by studies conducted in India, Asia, and Ethiopia [11, 12, 21]. Due to that, treatment with Zidovudine results in suppressions of bone marrow and other hematopoietic activities, which leads to low production of red blood cells and other types of blood cells in the bone marrow. Another mechanism of Zidovudine-induced anemia is mainly attributable to inhibition of proliferation of blood cell progenitor cells in a time and dose-dependent fashion [34–36].

### Limitation of the study

Since the data were collected from a secondary source of medical records, other important predictors of anemia, like income, food diversification, intestinal parasite infestations, maternal serum hemoglobin level, and serum ferritin level were not assessed. In addition, there might be selection bias because there were incomplete charts that were excluded from the analysis.

## Conclusion

The overall incidence rate of anemia was high compared to other country reports. Age, clinical, and ART-related variables provoked the incidence of anemia. Therefore, a need to emphasize the younger age group, prevent and manage opportunistic infections of WHO clinical stage III and IV, and select and monitor appropriate ART regimens.

### Recommendation

#### To policymakers

It is strongly advised to strengthen a good ART adherence level. Optimum adherence is highly essential for sustainable success to highly active antiretroviral treatment because ART drug has an effect on preventing anemia among clients living with HIV/AIDS taking ART drugs.

#### To clinicians

Give special focus on identified predictors of anemia incidence in this study. Especially children who started ART with Zidovudine-based ART regimen, 0.25—≤ 5 years, WHO clinical stage III & IV, fair/Poor ART adherence level, and for those children being underweight to reduce the incidence rate of anemia among children on ART.

#### To future researchers

A prospective study design is recommended to identify additional predictors of anemia among children on ART like food diversification, maternal serum hemoglobin level, and serum ferritin level, which are found by the primary data source.

## Data Availability

The datasets generated during the current study are not publicly available due to confidentiality issues since the study was conducted among HIV-infected children. But data will be available upon reasonable request from the corresponding author.

## Abbreviations

AHR: Adjusted Hazard Ratio
AIDS: Acquired Immunodeficiency Syndrome
ART: Antiretroviral Therapy
AZT: Zidovudine
BMI: Body Mass Index
CD4: Cluster of Differentiation 4
CHR: Crude Hazard Ratio
CI: Confidence Interval
CPT: Cotrimoxazole Prophylactic Therapy
EDHS: Ethiopian Demographic Health Survey
HAART: Highly Active Antiretroviral Therapy
HIV: Human Immunodeficiency Virus
HGB: Hemoglobin
IRB: Institutional Review Board
OI: Opportunistic Infection
PCV: Packed Cell Volume
RBC: Red Blood Cell
TB: Tuberculosis
WHO: World Health Organization

## References

[1] Ebonyi AO, Oguche S, Ochoga MO, Agbaji OO, Anejo-Okopi JA, Abah IO (2017). Changes in the hematological parameters of HIV-1 infected children at 6 and 12 months of antiretroviral therapy in a large clinic cohort, North-Central Nigeria. J Virus Erad.

[2] Ezeonwu B, Ikefuna A, Oguonu T, Okafor H (2014). Prevalence of hematological abnormalities and malnutrition in HIV-infected under-five children in Enugu. Niger J Clin Pract.

[3] Geletaw T, Tadesse MZ, Demisse AG (2017). Hematologic abnormalities and associated factors among HIV infected children pre-and post-antiretroviral treatment, North West Ethiopia. Journal of blood medicine.

[4] Oshikoya K, Lawal S, Oreagba I, Awodele O, Olayemi S, Iroha E (2012). Adverse events in HIV-infected children on antiretroviral therapy at a teaching hospital in Lagos, Nigeria: a retrospective study. Adv Pharmacoepidem Drug Safety.

[5] Ruhinda EN, Bajunirwe F, Kiwanuka J (2012). Anaemia in HIV-infected children: severity, types, and effect on response to HAART. BMC Pediatr.

[6] Woldie H, Kebede Y, Tariku A (2015). Factors associated with anemia among children aged 6–23 months were attending growth monitoring at Tsitsika Health Center, Wag-Himra Zone, Northeast Ethiopia. J Nutr Metab.

[7] Enawgaw B, Alem M, Melku M, Addis Z, Terefe B, Yitayew G (2015). Prevalence and associated risk factors of anemia among HIV-infected children attending Gondar university hospital, Northwest Ethiopia: a cross-sectional study. BMC Hematology.

[8] Renner LA, Dicko F, Koueta F, Malateste K, Gueye RD, Aka E (2013). Anaemia and zidovudine-containing antiretroviral therapy in pediatric antiretroviral programs in the IeDEA Paediatric West African Database to evaluate AIDS. J Int AIDS Soc.

[9] Ethiopian public Health institute. Central Statistical Agency EaIEdahs. Key Indicators Report. In Addis Ababa, Ethiopia, EDHS 2016,pdf. 2016.

[10] Wagnew F, Eshetie S, Alebel A, Tesema C, Kibret GD, Gebrie A (2019). Burden of anemia and its association with HAART in HIV infected children in Ethiopia: a systematic review and meta-analysis. BMC Infect Dis.

[11] Bunupuradah T, Kariminia A, Chan K-C, Ramautarsing R, Huy BV, Han N (2013). Incidence and predictors of severe anemia in Asian HIV-infected children using first-line antiretroviral therapy. Int J Infect Dis.

[12] Techane MA, Anlay DZ, Tesfaye E, Agegnehu CD (2020). Incidence and predictors of anemia among children on antiretroviral therapy at the University of Gondar Comprehensive Specialized Hospital, Northwest Ethiopia, 2007–2017: a retrospective follow-up study. HIV/AIDS (Auckland, NZ).

[13] Obirikorang C, Yeboah FA (2009). Blood hemoglobin measurement as a predictive indicator for the progression of HIV/AIDS in a resource-limited setting. J Biomed Sci.

[14] World Health Organization. Antiretroviral therapy of HIV infection in infants and children: towards universal access: recommendations for a public health approach-2010 revision. World Health Organization; 2010. ISBN 9241599804. 23741772

[15] Sauvageot D, Schaefer M, Olson D, Pujades-Rodriguez M, O’Brien DP. Antiretroviral therapy outcomes in resource-limited settings for HIV-infected children< 5 years of age. Pediatrics. 2010;125(5):e1039–47. 10.1542/peds.2009-1062 20385636

[16] Prendergast A, Walker AS, Mulenga V, Chintu C, Gibb DM (2011). Improved growth and anemia in HIV-infected African children taking cotrimoxazole prophylaxis. Clin Infect Dis.

[17] Melku M, Enawgaw B, Ayana S, Anlay DZ, Kebede A, Haile A (2020). Magnitude of anemia and undernutrition among HIV-infected children who took HAART: a retrospective follow-up study. Am J Blood Res.

[18] Adane A, Desta K, Bezabih A, Gashaye A, Kassa D (2012). HIV-associated anemia before and after initiation of antiretroviral therapy at Art Centre of Minilik II Hospital, Addis Ababa. Ethiopia Ethiop Med J.

[19] Daka D, Lelissa D, Amsalu A (2013). Prevalence of anemia before and after the initiation of antiretroviral therapy at ART center of Hawassa University Referral Hospital, Hawassa. South Ethiopia Sch J Med.

[20] Mihiretie H, Taye B, Tsegaye A (2015). Magnitude of anemia and associated factors among pediatric HIV/aids patients attending Zewditu memorial hospital art clinic, Addis Ababa, Ethiopia. Anemia.

[21] Rajesh R, Vidyasagar S, Varma DM, Mohiuddin S (2011). Evaluation of incidence of Zidovudine induced anemia in Indian human immunodeficiency virus-positive patients in comparison with stavudine based highly active antiretroviral therapy. Int J Risk Saf Med.

[22] Asrie F, Bazezew A, Motbaynor A, Zeleke B, Dessie K, Bimrew S, et al. Magnitude of anemia and associated factors among human immunodeficiency virus-infected children on highly active antiretroviral therapy at University of Gondar Comprehensive and Specialized Referral Hospital Northwest Ethiopia. Clinical Laboratory. 2020;66(6). 10.7754/Clin.Lab.2019.190835 32538059

[23] Shet A, Mehta S, Rajagopalan N, Dinakar C, Ramesh E, Samuel N (2009). Anemia and growth failure among HIV-infected children in India: a retrospective analysis. BMC Pediatr.

[24] World Health Organization. Hemoglobin concentrations for the diagnosis of anemia and assessment of severity. Vitamin and Mineral Nutrition Information System. Geneva: 2011. World Health Organization (WHO/NMH/NHD/MNM/111). 2011.

[25] The Federal Democratic Republic of Ethiopia Ministry of Health, National Guidelines for HIV/AIDS and Nutrition; 2008.

[26] National consolidated guidelines for comprehensive HIV prevention, care and treatment. 2018 [cited dec1,2021]. Available from: https://www.afro.who.int/sites/default/files/201904/National%20Comprehensive%20HIV%20Care%20%20Guideline%202018.pdf.

[27] Federal Ministry of health. National Comprehensive HIV Care and Treatment Training for Health care Providers Participant Manual June 2014.

[28] Kejo D, Petrucka PM, Martin H, Kimanya ME, Mosha TC (2018). Prevalence and predictors of anemia among children under five years of age in Arusha District, Tanzania. Pediatric health, medicine, and therapeutics.

[29] Tine RC, Ndiaye M, Hansson HH, Ndour CT, Faye B, Alifrangis M (2012). The association between malaria parasitemia, erythrocyte polymorphisms, malnutrition and anemia in children less than ten years in Senegal: a case-control study. BMC Res Notes.

[30] Wang J, Wang H, Chang S, Zhao L, Fu P, Yu W (2015). The influence of malnutrition and micronutrient status on anemic risk in children under 3 years old in poor areas in China. PLoS One.

[31] Beletew B, Mengesha A, Ahmed M, Fitwi A, Wudu M. Determinants of anemia among HIV-positive children on highly active antiretroviral therapy attending Hospitals of North Wollo Zone, Amhara Region, Ethiopia, 2019: a case-control study. anemia. 2020;2020. 10.1155/2020/3720572 PMC7049326 32148954

[32] Gebremedhin KB, Haye TB. Factors associated with anemia among people living with HIV/AIDS taking ART in Ethiopia. Advances in hematology. 2019;2019. 10.1155/2019/9614205 PMC6421011 30941180

[33] Dachew BA, Tesfahunegn TB, Birhanu AM (2014). Adherence to highly active antiretroviral therapy and associated factors among children at the University of Gondar Hospital and Gondar Poly Clinic, Northwest Ethiopia: a cross-sectional institutional-based study. BMC Public Health.

[34] Ejeliogu EU, Oguche S, Ebonyi AO, Okpe SE, Yiltok ES, Ige O (2014). Zidovudine-induced anemia in human immunodeficiency virus-infected children on highly active antiretroviral therapy in Jos, Nigeria.

[35] Dash KR, Meher LK, Hui P, Behera S, Nayak S (2015). High incidence of Zidovudine induced anemia in HIV infected patients in Southern Odisha. Indian J Hematol Blood Transfus.

[36] Chatterjee A, Bosch RJ, Kupka R, Hunter DJ, Msamanga GI, Fawzi WW (2010). Predictors and consequences of anemia among antiretroviral-naive HIV-infected and HIV-uninfected children in Tanzania. Public Health Nutr.

